# Insights Into the Role of DNA Methylation in Immune Cell Development and Autoimmune Disease

**DOI:** 10.3389/fcell.2021.757318

**Published:** 2021-11-01

**Authors:** Jiaqi Li, Lifang Li, Yimeng Wang, Gan Huang, Xia Li, Zhiguo Xie, Zhiguang Zhou

**Affiliations:** ^1^National Clinical Research Center for Metabolic Diseases, Key Laboratory of Diabetes Immunology (Central South University), Ministry of Education, and Department of Metabolism and Endocrinology, The Second Xiangya Hospital of Central South University, Changsha, China; ^2^Department of Ultrasound, The Third Xiangya Hospital of Central South University, Changsha, China

**Keywords:** DNA methylation, T/B cell development and differentiation, cell memory, autoimmune diseases, DNA methyltransferases

## Abstract

To date, nearly 100 autoimmune diseases have been an area of focus, and these diseases bring health challenges to approximately 5% of the population worldwide. As a type of disease caused by tolerance breakdown, both environmental and genetic risk factors contribute to autoimmune disease development. However, in most cases, there are still gaps in our understanding of disease pathogenesis, diagnosis, and treatment. Therefore, more detailed knowledge of disease pathogenesis and potential therapies is indispensable. DNA methylation, which does not affect the DNA sequence, is one of the key epigenetic silencing mechanisms and has been indicated to play a key role in gene expression regulation and to participate in the development of certain autoimmune diseases. Potential epigenetic regulation via DNA methylation has garnered more attention as a disease biomarker in recent years. In this review, we clarify the basic function and distribution of DNA methylation, evaluate its effects on gene expression and discuss related key enzymes. In addition, we summarize recent aberrant DNA methylation modifications identified in the most important cell types related to several autoimmune diseases and then provide potential directions for better diagnosing and monitoring disease progression driven by epigenetic control, which may broaden our understanding and contribute to further epigenetic research in autoimmune diseases.

## Introduction

Autoimmune diseases, which represent a family of almost 100 conditions, have received mounting and widespread attention due to their complex etiologies and the life-long threat they pose. The initial study of autoimmune disease prediction can be traced back to the late 20th century and demonstrated that early risk factors for inducing autoimmune responses exist in the genes of the major histocompatibility complex (MHC) (Weetman and McGregor, 1984). The etiology of autoimmune diseases is multifactorial. In addition to variants in immune genes and environmental factors, certain internal factors, including sex, age, and mental and emotional status, can also affect autoimmune responses, thus changing the possibility of developing clinical disease. The incidence rate of autoimmune diseases is high in industrialized countries, and females are predominantly affected, which is partially due to parent-of-origin differences in DNA methylation of the X chromosome (Golden et al., 2019). Although the clinical characteristics are diverse, all of these diseases have a basic etiology: a self-reactive adaptive immune response in which many lymphocytes participate (Rose, 2016) and a break of immune tolerance is the main character. Since most autoimmune diseases may have caused severe tissue damage before clinical diagnosis, it is necessary to make efforts to diagnose and treat them as soon as possible before irreversible damage occurs (Christen, 2019).

Epigenetics refers to heritable changes in gene expression separate from the DNA sequence that are mediated through a series of mechanisms regulated by environmental signals (Zheng et al., 2008). The major epigenetic regulation mechanisms include DNA methylation, histone modification and non-coding RNA regulation. To date, several lines of evidence confirm the important functions of epigenetic modifications in autoimmune diseases, especially DNA methylation, shedding light on disease pathogenesis, progression and activity to a certain extent (Wang et al., 2015).

In this review, DNA methylation, one of the major epigenetic adjustment mechanisms, will be reviewed, with particular attention on the function of DNA methylation in the types of cells involved in autoimmune diseases, the genomic methylation patterns involved in differentiation/development events and the dysregulated immune responses in specific autoimmune diseases. Moreover, the potential for epigenetic regulators as biomarkers and therapeutics for these diseases will be discussed.

## Overview of DNA Methylation

**FIGURE 1:**
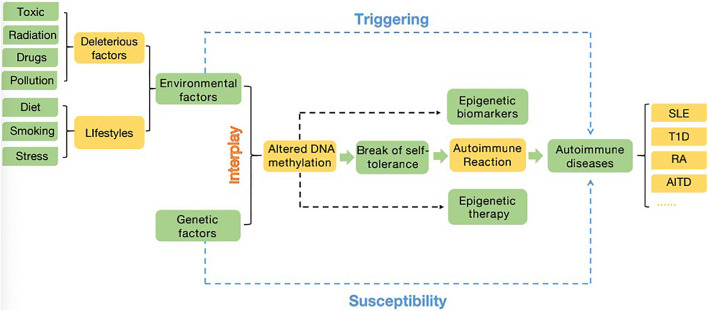
A close interplay between the environment (deleterious factors and lifestyles) and genetic factors (related genes, SNPs, copy number variation…), the altered DNA methylome is responsible for the break of self-tolerance, leading to autoimmune reaction and then account for the emergence and/or progression of autoimmune diseases. The changed DNA methylated profiles can also provide new insights into diagnostic and therapeutic methods in autoimmune diseases. The major potential for epigenetics applying in clinical can be summarized as epigenetic biomarkers and epigenetic therapy. Additionally, environmental factors have ability to trigger autoimmune disease, while genetic factors provide susceptibility to autoimmune diseases (Zouali, 2021).

One of the earliest discovered (∼1969) and intensely studied epigenetic regulation mechanism is DNA methylation, which functions in producing heritable phenotypic changes without affecting the DNA sequence (Bird, 2002; Xie et al., 2018). Thus, unlike genetic changes, epigenetic aberrations are reversible, which provides a direction for disease treatments by pharmaceutically inhibiting dysregulated epigenetic regulation (Zhang et al., 2020). The spectrum and distribution of methylation levels and patterns can vary between populations. Both nematodes and the insect *Drosophila melanogaster* have been reported to lack methylation due to their undetectable m5C expression level and absence of DNA methyltransferases (DNMTs) (Gowher et al., 2000). In mammals, the majority of DNA methylation mainly occurs on cytosine-guanine dinucleotide (CpG) sites, and the percentage of methylated CpG sites in the human genome is 70%∼80%. However, evidence has shown that a level of methylation on non-CpG sites exists in mouse and human embryonic stem cells (ESCs) (Xie et al., 2012). A proportion of unmethylated CpG dinucleotides are enriched mainly in gene promoter regions and are always located in clusters called CpG islands (CGIs) (Husquin et al., 2018; Li et al., 2021). In addition, there are regions called CGIs shore that are located no more than 2 kb from CGIs, which have strongly conserved tissue-specific methylation patterns. The methylation of both CGIs and CpG shores is strongly related to gene expression reduction (Irizarry et al., 2009). Moreover, the pattern and level of DNA methylation are influenced by the complex interplay of environmental and genetic factors. For example, some deleterious factors including toxic, radiation, drugs, and pollution. Moreover, lifestyles such as diet (folate uptake), smoking and stress are also typical environmental factors. Additionally, viral or bacterial infection, inflammatory cytokines induction (Rui et al., 2016; Sanderson et al., 2019; Zouali, 2021; Figure 1). As one of the most important and well-known epigenetic mechanisms, DNA methylation has been proposed to be involved in gene expression regulation and cell differentiation by cooperating with other regulators (Klutstein et al., 2016), as well as in chromatin structure. Subsequent chromatin remodeling can affect the production of many key proteins required for the normal function of the immune system (Lal et al., 2009).

The DNA methylation process involves a chemical modification in which specific bases in the DNA sequence are catalyzed by DNMTs and S-adenosyl methionine (SAM) is recruited as a methyl donor to obtain a methyl group for 5mC formation via covalent bonding (Moore et al., 2013). DNA methylation plays a maintainable role during normal development and functions in gene repression through silent chromatin reconstruction during each round of replication (Cedar and Bergman, 2012). With development, a substantial portion of DNA methylation in the blastocyst is gradually removed, and an epigenetic ground state is formed. Then, a wave of *de novo* methylation is established during X-chromosome inactivation, and almost all CpGs in the genome are modified at that time except protected CGIs (Cedar and Bergman, 2012). This brings about gene silencing on the inactivated chromosome, and housekeeping genes are expressed in all cells. After stage- and/or tissue-specific methylation changes, the epigenetic patterns of each individual cell type are ultimately molded (Meng et al., 2015). The DNA sequence information leads to this change, which serves as an important functor in the aspect of long-term expression stability.

**FIGURE 2:**
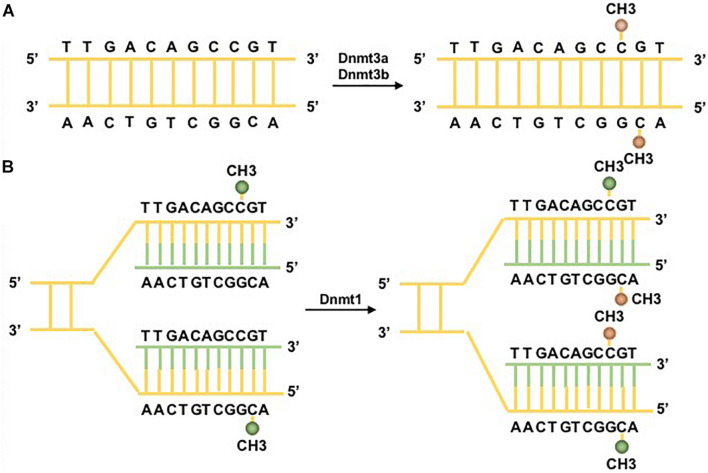
DNA methylation pathways. Two major DNA methyltransferases (DNMTs) participate in the formation of 5-methylcytosine (5mC). **(A)** DNMT3a/3b/3L are the *de novo* DNMTs and transfer methyl groups (yellow) onto naked DNA. **(B)** DNMT1 is the maintenance DNMT and plays roles in maintaining the DNA methylation pattern during replication. Under the situation of DNA semiconservative replication, the parental DNA strand retains the original DNA methylation pattern (green). DNMT1 links to replication foci and precisely replicates the original DNA methylation pattern by adding methyl groups (yellow) onto the newly formed daughter strand (green) (Moore et al., 2013).

**FIGURE 3:**
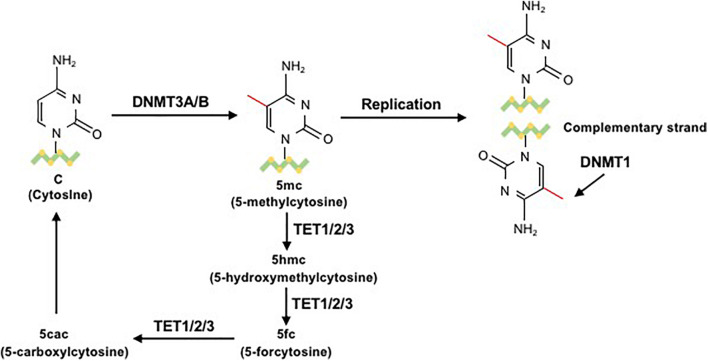
The process of DNA methylation and demethylation. In the presence of the cofactor S-adenosyl methionine (SAM), the unmodified fifth carbon of cytosine resides in the DNA sequence to form a 5mC methyl group through the action of DNMT3a/b. 5-Methylcytosine (5mC) is mainly located on CpG dinucleotides in somatic cells. During the replication process, DNMT1 methylates the daughter chain to maintain 5mC. 5mC can be sequentially oxidized to 5hmC, 5fC, or 5caC by ten-eleven translocation (TET) cytosine dioxygenase enzymes. Then, 5fC and 5caC can be converted to unmodified cytosine (Correa et al., 2020).

The reversible function of epigenetic modification is due to the presence of enzymes that catalyze the apposition of posttranslational regulation, including histone methyltransferases and histone acetylases, which are recognized as epigenetic writers, and enzymes that act in the demethylation and deacetylation of histones, which are considered as epigenetic erasers (Renaude et al., 2021). A group of DNMTs (DNMT3a, DNMT3b, and DNMT1 are dominant) function in the establishment and maintenance of DNA methylation patterns in mammals. Both DNMT3a and DNMT3b enable the construction of a new methylation pattern for unmodified DNA, which is essential for their roles in transferring methyl groups during *de novo* methylation (Feng et al., 2005). Evidence in mouse embryonic stem (ES) cells has shown that the genomic enrichment pattern of DNMT3a is not consistent with that of DNMT3b, which reveals a phenomenon in which each DNMT has specific targets reflecting their unique N-terminal domains during the development process. Genetic ablation of DNMT3a and DNMT3b leads to lethal phenotypes at different developmental stages. DNMT3a is required for establishing maternal imprints in differentially methylated regions (DMRs), and DNMT3b plays a leading role in inactivation of X chromosomes (Manzo et al., 2017; Yagi et al., 2020). DNMT1 is localized at replication foci and always acts in cell division. As a maintenance enzyme, DNMT1 crucially functions in preserving the stability of established DNA methylation patterns (Goll and Bestor, 2005; Figure 2). Furthermore, as an essential cofactor for *de novo* methyltransferase in ES cells, DNMT3L is highly expressed in ES and germ cells and plays a key role in the methyltransferase activity of DNMT3a and DNMT3b via a physical interaction (Ooi et al., 2010). Additionally, research has demonstrated that the methyltransferase activity of DNMT2 is weak *in vitro*, and deletion of DNMT2 has little effect on CpG methylation levels or developmental phenotypes (Goll and Bestor, 2005). Moreover, the harmony of the DNA methylation level requires balanced control between DNA methylation and demethylation. Replication-independent active DNA demethylation and replication-dependent passive DNA demethylation are two major pathways to reverse repressed gene expression. Ten-eleven translocation (TET) demethylases are key DNA demethylation enzymes (Lio and Rao, 2019). In addition, the existence of DNA methylation variability, which is due to polymorphisms or mutations in target genes, has the ability to influence the phenotype of an individual (Imgenberg-Kreuz et al., 2018). Aberrant methylation may serve as a risk factor for some autoimmune diseases and may be caused by the influence of aging or the environment (Figure 3).

## DNA Methylation in T Cell Development

T cells are regarded as key mediators in immunity and immunologic memory. Their differentiation fates can be partially regulated by epigenetic mechanisms, such as DNA methylation (Liu et al., 2019). Recently, genome-wide methylation analyses have demonstrated dynamic changes in the methylome during different development and differentiation processes and that some DNA regulators are involved in controlling various aspects, including cell fate decisions, function, and stability (Ji et al., 2010). Based on the fact that dysregulated T cells participate in different disease states, including autoimmune diseases, chronic inflammatory diseases and cancer, more detailed knowledge of how epigenomic programming functions in these pathologic states is critical (McLane et al., 2019; Correa et al., 2020).

### DNA Methylation in T Helper Cell Development and Function

While genetic and environmental factors are known risk factors for autoimmune diseases, incomplete disease concordance between identical twins supports the notion that other factors play a role in disease development and progression. Recently, convincing evidence has indicated that epigenetic modifications, particularly impaired T cell DNA methylation, contribute to this additional factor (Makar et al., 2003; Generali et al., 2017). Naïve CD4^+^ T cells are characterized by high plasticity and have the ability to differentiate into discrete lineages with unique functions in the immune response. Then, differentiated T helper (Th) cells can maintain their original lineage selection under the condition of stable transcriptional memory to resist redifferentiation (Thomas et al., 2012a). During thymic development, the interleukin 4 (IL4) expression of naïve CD4^+^ T cells has been reported to depend on epigenetic programming, which is consistent with the CD4/CD8 lineage. In the subsequent steps of peripheral maturation, mechanisms involving DNA methylation at the IL4-IL13 locus can partially suppress this IL4 expression potential (Makar et al., 2003). Transcriptional permission of the IL4–IL13 locus in naïve CD4^+^ T cells still exists and is not affected by the accumulation of repressive DNA methylation marks (Baguet and Bix, 2004). Moreover, the process by which Th cells differentiate into mature Th1 and Th2 fates is epigenetically regulated. DNMT1 plays an important role in repressing cytokine production, and depletion of DNMT1 mediated by CD4Cre brings about increased expression of the cytokines interferon-γ (IFN-γ), IL2, IL3, and IL4 in activated CD4^+^/CD8^+^ T cells and decreased proliferation of peripheral T cells (Lee et al., 2001). Th1 cells show an IFN-γ-demethylated promoter and act in fighting against bacteria by producing IFN-γ. As the key lineage marker for Th1 cells, the Ifng genomic locus is hypomethylated, and this pattern is maintained under Th1 polarized conditions *in vitro*, which is opposite to the conditions in Th2 cells, which show a hypermethylated Ifng locus and hypomethylated IL4 locus in CD4^+^ T cells (Santangelo et al., 2002). Furthermore, the disassociation of DNMT1 and effector cytokine IL4 loci is the crucial step for IL4 expression during Th2 differentiation (Makar et al., 2003). The conditions of low IL4 expression in Th2 cells can be changed using 5-azacytidine (a hypomethylating agent), which has demonstrated that the magnitude of cytokine production in CD4^+^ T cells can be regulated by the degree of DNA methylation (Guo et al., 2002). In contrast to DNMT1, *de novo* methylation mediated by DNMT3a is unable to affect the initial differentiation of Th1 and Th2 cells but is required to restrict Th lineage plasticity (Thomas et al., 2012a).

Epigenetics also participate in and provide molecular regulators for the progression of human naïve T cells (Tn) to differentiate into distinct types of memory cells and their long-term maintenance (Durek et al., 2016). Data from comprehensive epigenome and transcriptional analyses of the human CD4^+^ T cell population have shown that there is progressive DNA methylation loss during the transition from the naïve to memory stages. This loss of methylation tends to occur in “partially methylated domains (PMDs)” (Hon et al., 2012) and serves as a common characteristic in B cell differentiation. Moreover, evidence has shown that there is an association between PMDs and heterochromatic histone signatures, as well as regions replicated in late S phase, and gradually lose methylation during excessive proliferation (Aran et al., 2011). In addition, a dynamic change in methylation states also participates in the differentiation of CD4^+^ T cells to Th17 cells. Cooperation between DNA methylation and conserved intergenic elements contributes to control of transcription at the IL17 locus (Thomas et al., 2012b). DNMT3a is required for the stability of the Th17 program by suppressing the production of IFN-γ (Thomas et al., 2012a). Therefore, DNA methylation controls Foxp3 expression and plays an important role in T cell fate and function.

### DNA Methylation in Regulatory T Cells Development and Function

As a subset of CD4^+^ T cells, regulatory T cells (Tregs) play roles in limitation of inflammatory reactions and immune responses. As the “master regulator” of Tregs, Foxp3 expression is crucial for the development and function of Tregs and is present in the thymus in natural Tregs (nTregs) (Josefowicz et al., 2009; Li et al., 2014). Conserved non-coding sequence (CNS) 2, one of the major CNSs controlling Foxp3 expression, is made up of numerous CpG elements and is especially controlled by DNA methylation (Correa et al., 2020). Evidence has demonstrated that there is a unique and evolutionarily conserved CpG-rich island in the Foxp3 non-intronic upstream enhancer that is excessively methylated in conventional CD4^+^ T cells, activated CD4^+^ T cells, and peripheral TGF-β-induced Tregs but demethylated in nTregs (Floess et al., 2007). In addition to the Foxp3 locus, the establishment of a Treg cell-specific CpG hypomethylation pattern also led to Treg cell development in a Foxp3-independent manner (Ohkura et al., 2012). Notably, DNMT1 may provide a possibility for DNA methylation to act in maintaining suppression of Foxp3 in thymic and peripheral Foxp3-negative CD4^+^ T cells upon T cell receptor (TCR) stimulation. Although Tregs with DNMT1 deficiency are unable to change the methylation of CNS2 in Foxp3, global changes in DNA methylation are related to the deletion of several genes crucial to Treg function and an increase in inflammatory gene expression (Kim and Leonard, 2007; Polansky et al., 2008).

### DNA Methylation in T Cell Differentiation and Memory

DNA methylation is suitable for a particular cellular memory function in development due to its features of methylation state heritability and the secondary nature of the decision to include or exclude methylation (Radbruch et al., 2021). The epigenome and transcriptome of human CD4^+^ T cells suggests that progressive changes in DNA methylation loss exist in the memory development of CD4^+^ T cells, with a linear pattern in the order of Tn-T central memory (Tcm) – T effector memory (Tem) – T CD45RA^+^ memory (Temra), while tissue-resident bone marrow- long-lived memory (Tmem) cells branch off with a unique epigenetic profile (Durek et al., 2016). Based on the differential methylation spectrum, differentiated CD4^+^ memory cells can be distinguished, especially in the context of Th1 and T follicular helper (Tfh) committed cells (Hale et al., 2013). A study using TCR transgenic CD4^+^ T cells pointed out that gene-specific DMRs are positioned at related gene-enhancer regions and that these regions are related to different expression levels of memory-associated genes (Hashimoto et al., 2013); moreover, there is a similar situation in CD8^+^ T cells (Scharer et al., 2013).

As one of the most indispensable components of long-lived T cell immunity, there is still a long-standing debate centered on the formation of memory CD8^+^ T cells, while the specific mechanism by which memory CD8^+^ T cells retain naïve and effector characteristics remains unclear (Ahmed et al., 2009). A series of studies have demonstrated that genome-wide epigenetic reprogramming is involved in the differentiation of CD8^+^ T cells. Upon infection with LCMV-Armstrong, T cells experience dynamic DNA remodeling during the transition from naïve to effector CD8^+^ T cells. Moreover, related gene expression during this transition is negatively correlated with DNA methylation localized in proximal promoter regions. Both enhancer and gene promoter regions showing differential methylation are enriched for functional transcription factor motifs (Scharer et al., 2013). Recently, a study found that the coupled process of the inhibition of a naïve transcriptional programmer in memory precursor effector cells and *de novo* DNA methylation of the gene could be eliminated in a cell division-independent process due to the cells reacquiring re-expression of naïve-associated genes (Youngblood et al., 2017). Given the known understanding of *de novo* methyltransferase activity, DNMT3a serves as a critical director in early CD8^+^ T cell effector and memory fate commitments. Further, conditional deletion of DNMT3a has been found to promote the kinetics of memory cell development. One study showed that memory precursor cells could obtain *de novo* methylation programs mediated by DNMT3a at critical loci, and the obtained methylation programs could be erased, leading to re-expression of naïve genes during the development of memory CD8^+^ T cells (Youngblood et al., 2017). However, inconsistent with this report, another study found that terminal effectors obtain *de novo* programs at critical loci, while these *de novo* programs are absent in memory precursor cells. Furthermore, DNMT3a-deficient T cells prefer to produce more memory precursors and fewer terminal effector cells in a T-cell internal manner instead of enhancing the plasticity of differentiated effector CD8^+^ T cells. Additionally, DNMT3a depletion tends to differentiate early effector cells into memory precursor cells without *de novo* methylation programs (Ladle et al., 2016). All these results support the idea that DNA methylation functions in CD4/CD8^+^ T cell differentiation and memory.

## DNA Methylation in B Cell Development

B cells serve as essential actors in the initialization and acceleration of autoimmune diseases (Ceccarelli et al., 2016). Once mature naïve B cells migrate to the peripheral lymphoid system and are exposed to self- and/or foreign antigens, the corresponding antigen-specific B cells are activated through signals from Th cell-produced cytokines and the help of Tfh cells. Then, activated B cells differentiate into plasma cells or memory B cells by undergoing a series of processes, which provide humoral immune functions (Alt et al., 2013). Several lines of evidence have demonstrated that epigenetic regulation is involved in the somatic hypermutation (SHM) and class switch DNA recombination modifications under the condition of B cell activation and differentiation. Thus, any abnormal regulation involved in these processes may provide the possibility of aberrant antibody production and lead to the pathogenesis of autoimmune diseases (Wu et al., 2018). Therefore, it is essential to summarize current research progress in epigenetic regulation that promotes B cell activation and differentiation to better comprehend B cell biology and its role in autoimmune development.

### DNA Methylation in Germinal Center B Cells

The formation of germinal centers (GCs) is attributed to activated B cell proliferation under the promotion of cytokines originating from Th cells and Tfh cells (Alt et al., 2013). Rapid proliferation tolerance and the mutagenic actions of activation-induced cytosine deaminase (AICDA) are the typical phenotypes of GC B cells (Klein and Dalla-Favera, 2008). Based on the knowledge that DNA methylation patterns act as important regulators in determining cellular phenotypes (Wu et al., 2018), one study aimed to explore DNA methylation and the function of DNMTs in GC formation. The results from DNA methylation profiles reflected a significant shift in the DNA methylation pattern in GC B cells compared with resting/naïve B cells. Overall, 223 differentially methylated genes were involved and were relatively hypomethylated in GC B cells compared with resting/naïve B cells. Except for some B cell lineage genes, such as *Pax5, Ebf1, Cd19*, and *Spib*, which show a continuous active epigenetic status during B cell activation, almost all genome-wide DNA is hypomethylated. Moreover, greater DNA methylation heterogeneity was present in GC B cells, and the binding sites of AICDA were overexpressed at hypomethylated loci. The genes showing differential methylation predominately represent components of NF-κB and MAP kinase signaling. Accumulated evidence has suggested that differentially methylated genes are related to specific biological functions, such as metabolic regulation, and synthase, synthetase, chaperone and transporter enrichment. Additionally, the results revealed that DNMT1 was the only DNMT that was significantly upregulated in GC B cells. An animal study found that DNMT1 hypermorphic mice exhibit GC formation deficiency; once mice were treated with the DNMT inhibitor decitabine, GCs were unable to form after stimulation (Shaknovich et al., 2011). Interestingly, evidence from GC B cells of DNMT1 hypomorphic animals has demonstrated the dual effects of DNMT1 in DNA methylation and break repair of double-stranded DNA (Shaknovich et al., 2011). Furthermore, epigenetic regulation, including DNA methylation and histone modification, plays an important regulator at the SHM stage involved in B cell activation, which targets V(D)J DNA via transcription (Cui et al., 2016). Notably, the fact that a demethylated allele is the only allele that can be hypermutated in comparable transcription of both alleles further suggests a critical role for DNA methylation in SHM (Odegard and Schatz, 2006).

### DNA Methylation in B Cell Memory

Memory formation serves as a critical hallmark of adaptive immunity. In addition to T cells, epigenetic regulation also contributes to the differentiation of memory B cells. A series of studies have suggested that another epigenetic modification, histone modification, plays an important role in this process, for example, by controlling the hallmark genes of memory B cells, such as CD27 in humans and CD38 in mice (Zan and Casali, 2015), and can also inhibit *Irf4* and *Prdm-1* transcription by catalyzing H3K27me3, thereby regulating the percentage of memory B cells, GC reactions and antibody responses (Good-Jacobson, 2014).

To begin to comprehend how DNA methylation acts in the formation of memory B cells, one study has shown that a large proportion of DNA methylation loss induced by activation is mapped to transcription factor binding sites. An extra level of demethylated loci mapped to *Alu* elements, with the help of the genome and coexisting DNMT3a suppression. Activation-dependent DNA methylation changes in the offspring of activated B cells contribute a comparable epigenetic characteristic to downstream memory B cells and plasma cells with diverse transcriptional programs (Lai et al., 2013). These results revealed the methylation dynamics of the genome during cellular differentiation in an immune response.

## DNA Methylation in Autoimmune Diseases

The fundament of autoimmunity is self-tolerance. Although there is a growing body of research exploring the immune regulation related to autoimmunity, the specific mechanism that results in tolerance loss remains difficult to elucidate (Shoenfeld et al., 2008). Given that concordance rates in monozygotic (MZ) twins are no more than 50%, it is reasonable to speculate that there are other complementary mechanisms that participate in gene expression regulation, which eventually leads to dominant autoimmunity (Hewagama and Richardson, 2009; Meda et al., 2011). Additionally, whether in clinical settings or experimental models, an increasing number of studies have demonstrated that the epigenome is a critical actor in better understanding the initiation and perpetuation of autoimmunity (Meda et al., 2011).

Currently, an increasing number of studies have aimed to explore the effect of epigenetics in complicated disorders and to improve understanding of its distinct function within the field of medicine. Some hypotheses have noted that epigenetic modification, including DNA methylation, is considered a bridge connecting environmental stimulation and genetic factors in the pathogenesis of autoimmune diseases (Dupont et al., 2009).

Furthermore, the development of immune cells serves as a well-defined process in which progenitor cells produce progeny cells through a given differentiation pathway. The correctness of this process of differentiation and lineage commitment guarantees the establishment of immune tolerance. Thus, as one of the key regulators in immune cell differentiation and development, specific impairments in DNA methylation profiles could result in immune cell autoreactivity and predispose an individual to autoimmune dysregulation and risk for autoimmune diseases (Wang et al., 2015; Cumano et al., 2019). There is a relationship between DNA methylation defects and autoimmune disease pathogenesis. A genome-wide DNA methylation study quantified more than 4485,00 methylation sites across the genome (Coit et al., 2013). Thus, understanding the aberrant expression of DNA methylation mediators is critical for deciphering concurrent epigenetic alterations in various autoimmune diseases and for the development of new therapeutic strategies. In this section, we focus on the common autoimmune diseases systemic lupus erythematosus (SLE), type 1 diabetes (T1D), rheumatoid arthritis (RA), Graves’ disease (GD), and Hashimoto’s disease (HD), with the aim of clarifying the role of DNA methylation in disease pathogenesis and development (Table 1).

**TABLE 1:** Available evidence on DNA methylation changes involved in SLE, T1D, and other common autoimmune diseases.

	Specific target	Types of cells	Main findings	References
	ERK pathway signaling Methylation-sensitive autoimmune genes	Lupus T cells	ERK pathway signaling and chromatin structure impairments LFA1, *CD70 (TNFSF7)*, *CD40LG (TNSF5)*, *CD11a (ITGAL)*, perforin (*PRF1*) ↑ DNMT1 ↓	Gorelik and Richardson, 2009; Relle et al., 2015
	*IFI44L*	Whole blood	*IFI44L* promotor methylation ↓	Zhao et al., 2016
*SLE*	*PP2Ac*α	SLE T cells	DNMT1 ↓ *PP2Ac*α promotor hypomethylation, *PP2Ac*α↑	Sunahori et al., 2011
	Type 1 IFN-related genes	PBMCs	*IFIT1, IFI44L, MX1, RSAD2, OAS1, EIF2AK2*, and *NLRC5* are associated with autoantibody positivity	Yang et al., 2017
	T1D-MVPs	Purified CD14^+^ monocytes	58 hypermethylated and 74 hypomethylated genes, typically *HLA-DQB1, GA62, TNF*, and *TRAF6*	Rakyan et al., 2011; Cerna, 2019
	T1D-associated DNA methylation profiles	EBV immortalized B cells	88 significant changes at CpG sites, typically in *HLA-E, HLA-DOB, HLA-DQ26, INS, IL2RB*, and *CD226*	Stefan et al., 2014
	MHC region and T1D-associated CpG sites	Peripheral blood	Mostly methylation of *MAGI2, FANCC* and *PCDHB16* Modest methylation of *BACH2, INS-IGF2*, and *CLEC16A*	Elboudwarej et al., 2016
*T1D*	T1D-related MVPs	CD4^+^ T cells; CD19^+^ B cells; CD14^+^CD16^–^monocytes	T1D-related MVPs positioned at genes involved in immune cell metabolism and cell cycle, including mTOR signaling	Paul et al., 2016
	*IL2RA*	Whole blood cells (WBCs)	*IL2RA* promoter is associated with methylation of CpG site; 349 differential CpG methylation sites in T1D patients with PDR and without PDR; 19 potential CpG sites associated with the risk of T1D-related DR	Bell et al., 2010; Belot et al., 2013; Agardh et al., 2015
	Human *IGFBP1* gene	Whole peripheral blood cells	DNA methylation levels in the *IGFBP1* gene ↓; circulating IGFBP-1 levels in T1D patients ↑	Gu et al., 2014
		T cells and monocytes	Global hypomethylation	Nakano et al., 2013
	Genome-wide DNA methylation profiles	CD19^+^ B cells, synovial fibroblasts and PBMCs	An altered pattern of DNA methylation and reduced 5mC expression *CD1C, TNFSF10, PARVG, NID1, DHRS12, ITPK1, ACSF3*, and *TNFRSF13C* are signatures in SLE patients	Ai et al., 2018; Rodriguez-Ubreva et al., 2019
*RA*	*CD40L*	T cells	CD40L promoter demethylation in silenced X chromosomes caused CD40L overexpression, which plays a role in RA development	Lu et al., 2007
	*IL2RA (CD25)* and *CTLA-4*	Treg cells	SNPs of *IL2RA (CD25)* and *CTLA-4* are associated with RA susceptibility, and their aberrant DNA methylation pattern affect Foxp3 reactivation and impair the normal function of Treg cells	Ohkura and Sakaguchi, 2020
*GD and HD*	27728 annotated CGIs and 22532 promoters	Peripheral blood cells	132 hypermethylated and 133 hypomethylated regions in GD patients *ADRB2, B3GNT2, PADI4, TNFRAF25 (DR-3), ICAM1, MECP2*, and DNMT1 are regulated by DNA methylation and involved in GD development	Cai et al., 2015; Guo et al., 2018
	*IL6*	Peripheral blood cells	*IL6* methylation level is related to GD intractability and HD susceptibility	Hirai et al., 2019
	DNA methylation-related genes		*DNMT, MTHFR*, and *MTRR* are related to AITD risk	Cai et al., 2016

### DNA Methylation in Systemic Lupus Erythematosus

Systemic lupus erythematosus is a multiorgan autoimmune disease characterized by the presence of an autoantibody to nuclear and/or cytoplasmic antigens. Abnormal differentiation and activation of immune cells induced by factors associated with genetic susceptibility and epigenetic modification play an unequivocal role in SLE etiology (Miao et al., 2014). In recent years, it has been increasingly appreciated that abnormal DNA methylation is involved in the pathophysiology of SLE, and one view suggests that DNA hypomethylation and demethylated DNA fragments may influence the structure of T cell chromatin, leading to cellular hyperactivity and inducing the production of anti-DNA antibodies, thereby participating in the pathogenesis of SLE (Meda et al., 2011; Miao et al., 2014). A study in lupus T cells revealed that altered T cell DNA methylation in SLE is regulated by the extracellular signal-regulated kinase (ERK) signaling pathway. Furthermore, impairment of the ERK signaling pathway predominantly leads to SLE susceptibility in females, which is supported by a study in which only female mice with ERK impairment showed SLE-like symptoms versus male mice under the same conditions (Strickland et al., 2012). In murine models, this pathway is decreased, which leads to overexpression of methylation-sensitive autoimmune genes and downregulation of DNMT1 expression (Gorelik and Richardson, 2009). In addition, similar conclusions were also found in CD4^+^ T cells, CD19^+^ B cells, CD14^+^ monocytes, and neutrophils from SLE patients. Several methylation-sensitive genes were found to be hypomethylated in CD4^+^ T cells, such as lymphocyte function-associated antigen-1 *(LFA1*), *CD70 (TNFSF7)*, *CD11a* (integrin alpha L, *ITGAL)*, *CD40 ligand (TNFSF5)*, and *perforin (PRF1)*, leading to overexpression, similar to that observed in CD8^+^ T cells. All of these genes have a positive correlation with lupus disease activity. Furthermore, when compared with patients with inactive lupus and healthy individuals, the promoter regions of the genes mentioned above seem to be significantly hypomethylated in active lupus T cells (Richardson et al., 2012; Relle et al., 2015). Moreover, the promoter methylation of *IFI44L*, which is a blood biomarker for monitoring activity changes in SLE, has the ability to distinguish SLE patients from healthy controls with high sensitivity and specificity (Zhao et al., 2016). The use of an inhibitor of DNA methylation can result in hypomethylation of genes at the promoter region, and the corresponding genes are significantly upregulated (Sawalha et al., 2008). Another study using bisulfite sequencing showed a novel methylation-sensitive gene, serine/threonine-protein phosphatase 2A catalytic subunit α (*PP2Ac*α), which is induced by oxidative stress, shows increased expression in SLE T cells and contributes to the pathogenesis of SLE. Mechanistically, CpG methylation occurs in the cAMP response element (CRE) motif, which ultimately results in hypomethylated expression of the activity of the *PP2Ac*α promoter (Sunahori et al., 2011; Deng et al., 2019). Notably, an association between DNA methylation of type 1 IFN-related genes and autoantibody positivity has been identified in SLE. One study found that female SLE patients with and without a history of anti-dsDNA antibody positivity exhibit differentially methylated profiles (Yang et al., 2017). Furthermore, CD40L overexpression and corresponding demethylated genes on the inactive X chromosome are thought to be responsible for the female bias observed in SLE (Hewagama et al., 2013). On the other hand, the interplay between DNA methylation and microRNAs (miRNAs) in SLE has also been explored. Evidence has shown that the status of DNA methylation is regulated by some lupus-related miRNAs via targeting of DNA methylation enzymes or proteins associated with methylation pathways, such as genetic imprinting of Dlk1-Dio3 miRNAs (Lu et al., 2007; Dai et al., 2021).

### DNA Methylation in Type 1 Diabetes

Type 1 diabetes is a chronic, immune-mediated complex disorder caused by destruction of islet β cells that results in insulin deficiency, and both genetic and environmental factors are contributors to the pathogenesis of T1D (Xie et al., 2014, 2018; American Diabetes Association, 2020). A series of mechanisms linked to epigenetic regulation have been suggested to be involved in the development of T1D. One of the major mechanisms is regulation of lymphocyte maturation and cytokine gene expression, particularly for the differentiation of Th cell subtypes, which is regarded as the most complex immune process controlled by epigenetic regulation. Studies of genome-wide DNA methylation suggest that both dysregulated autoimmunity and primitive pancreatic damage are associated with abnormal DNA methylation (Xie et al., 2014; Elboudwarej et al., 2016; Paul et al., 2016). MZ twins are always employed to investigate the effect of epigenetic factors on disease development due to their almost identical genetic background and environmental exposures. A genome-wide DNA methylation profile for which purified CD14^+^ monocytes were collected from 15 MZ twins with discordant T1D onset identified 132 T1D-related methylation variable positions (T1D-MVPs), consisting of 58 hypermethylated and 74 hypomethylated MVPs. The strongest T1D susceptibility genes *HLA-DQB1* and *GA62* (encodes GAD65), the T1D-related inflammatory cytokine *TNF* and the TLR receptor signaling pathway-related protein *TRAF6* are representative MVPs found in this analysis; additionally, some of these MVPs were found to be altered prior to overt T1D onset and maintained temporal stability over many years, which may provide a potential possibility of early clinical diagnosis of T1D (Rakyan et al., 2011; Cerna, 2019). Similar studies have explored the DNA methylation patterns between MZ twins with discordant T1D onset and MZ twins with concordant T1D in Epstein Barr virus (EBV)-immortalized B cells (Stefan et al., 2014), peripheral blood (Elboudwarej et al., 2016), CD4^+^ T cells, CD19^+^ B cells, CD14^+^CD16^–^ monocytes (Paul et al., 2016), whole blood cells (Belot et al., 2013), whole peripheral blood and CD14^+^ monocytes (Cepek et al., 2016). The main findings and/or significantly changed CpG sites in all MZ twin pairs discordant for T1D are shown in Table 1. In addition, decreased immune tolerance is regulated by DNA methylation, which was found in CD4^+^ T cells from latent autoimmune diabetes in adults (LADA) and peripheral blood mononuclear cells (PBMCs) from fulminant type 1 diabetes (FT1D) (Wang et al., 2013; Agardh et al., 2015). Collectively, these findings further help to characterize the T1D risk conferred by the information encoded by the DNA methylome, which supports the notion that alterations in DNA methylation are involved in the pathogenesis of T1D.

Furthermore, a growing number of studies have supported the association between DNA methylation and diabetes complications in T1D patients, such as diabetic nephropathy (DN) and proliferative diabetic retinopathy (PDR) (Gu et al., 2014; Agardh et al., 2015). A study on T1D and DN revealed that 19 potential CpG sites are associated with DN risk, including one CpG site localized in *UNC13B*, which itself is related to DN (Agardh et al., 2015). A similar study also identified some CpG sites that are involved in transcription regulation and are related to DR risk (Bell et al., 2010). Based on the fact that the serum concentration of insulin-like growth factor binding protein-1 (IGFBP-1) is correlated with T1D, one study first found downregulated methylation of the *IGFBP1* gene in T1D patients, and T1D patients with DN showed a higher concentration of IGFBP-1 than the other two groups (Gu et al., 2014). In addition, another study focused on T1D and PDR showed that CpG sites with hypomethylation accounted for approximately 80% of the differentially methylated CpG sites found in T1D patients with PDR, which predicts that DNA methylation may be a potential biomarker for T1D with PDR. A series of studies have shown that these T1D-related MVPs are often positioned at gene regulatory elements of genes engaged in the immune cell cycle, cell metabolism and immune and defense responses (Stefan et al., 2014; Paul et al., 2016). Generally, these results support the idea that epigenetic modification plays a functional role in the pathogenesis of T1D.

### DNA Methylation in Rheumatoid Arthritis, Graves’ Disease, and Hashimoto’s Disease

Similar to SLE, RA is also recognized as a common autoimmune disease influenced by epigenetic regulation. Aberrant epigenomes, including DNA methylation, influence a series of inflammatory and matrix-related pathways and contribute to the pathogenesis of RA. Multiple studies have demonstrated that RA patients show global T cell and monocyte hypomethylation; an altered pattern of DNA methylation in CD19^+^ B cells, synovial fibroblasts and PBMCs; and reduced 5mC expression in synovial tissues compared with healthy controls (Nakano et al., 2013; Ai et al., 2018; Rodriguez-Ubreva et al., 2019; Fang et al., 2021). These hypomethylated genes are enriched in crucial ways associated with cell migration (Nakano et al., 2013). Furthermore, some of these genes, such as *CD1C, TNFSF10, PARVG, NID1, DHRS12, ITPK1, ACSF3*, and *TNFRSF13C*, also show a differentiated methylation signature in SLE patients (Ballestar et al., 2020; Fang et al., 2021). In addition, promoter region demethylation of *CD40L* in silenced X chromosomes leads to *CD40L* overexpression, which plays a role in RA development (Lu et al., 2007). Moreover, *IL2RA (CD25)* and *CTLA-4* are associated with RA susceptibility. As important Treg signature genes, single nucleotide polymorphisms (SNPs) in Treg-specific demethylated DNA regions of these two genes can affect Foxp3 reactivation and thereby impair the normal function of Treg cells (Ohkura and Sakaguchi, 2020).

DNA methylation modification is also a possible mechanism providing novel insight into autoimmune thyroid diseases (AITDs), which include Graves’ disease (GD) and Hashimoto’s disease (HD). Recently, attention has been given to the significance of DNA methylation in GD. A genome-wide methylation analysis covering 27728 annotated CGIs and 22532 promoters in peripheral blood cells uncovered an altered DNA methylation profile in GD patients, including 132 hypermethylated and 133 hypomethylated regions. Moreover, known candidate genes that were previously identified in GD or other autoimmune diseases were also found, such as *ADRB2, B3GNT2, PADI4, TNFRAF25 (DR-3)*, *ICAM1*, *MECP2*, and *DNMT1*, all of which are regulated by DNA methylation and involved in the development of GD (Cai et al., 2015; Guo et al., 2018). Moreover, another study showed that methylation levels of the *IL6* gene are linked to the intractability of GD and to susceptibility to HD (Hirai et al., 2019). In addition, a relationship between polymorphisms of genes involved in DNA methylation [such as *DNMT*, methylenetetrahydrofolate reductase *(MTHFR)* and methionine synthase reductase (*MTRR*)] and AITD risk has been demonstrated (Cai et al., 2016; Coppede, 2017).

## Conclusion and Perspectives

Over the past years, a large number of studies have explored the epigenetic regulation patterns that occur during the development of autoimmunity. As one of the three major epigenetic regulation patterns, the potential epigenetic modifications caused by DNA methylation have garnered more attention in recent years. Significantly, epigenetics can provide new insights into diagnostic and therapeutic methods for autoimmune diseases. The major potential for epigenetic application in the clinic can be summarized as epigenetic markers and epigenetic therapy. A series of epigenetic biomarkers, especially those associated with DNA methylation, are associated with clinical outcomes and provide an alternate stability profile rather than conventional testing based on DNA and RNA sequencing (Garcia-Gimenez et al., 2017; Table 1). Samples of blood, tissue, body fluid and secretions can be used to detect epigenetic biomarkers at the early stage of disease, which provides superiority compared with testing that is dependent on RNA and protein abnormalities (Zhang et al., 2020). On the other hand, a great deal of attention has been focused on epigenetic therapy, which is a novel option for disease treatment that employs epigenetic drugs or non-medical clinical management. For example, the first epigenetic drugs, azacytidine (5-AZA) and decitabine (5-AZA-CdR), were approved for clinical application in 2004 and have gradually been utilized for therapy targeting hematologic malignancies (Egger et al., 2004). Furthermore, a large number of epigenetic modifiers have been developed, and these modifiers can reprogram and reshape epigenetic patterns by reducing the level of DNA methylation and generating or removing epigenetic markers; thus, allowing full use of them would contribute to the treatment of diseases (Ballestar et al., 2020).

Although many lines of evidence have demonstrated that DNA methylation plays indispensable roles in autoimmune diseases by regulating immune cell differentiation and function, the specific mechanism by which it participates in the pathogenic states of autoimmune diseases still needs to be explored. In this review, we described DNA methylation to clarify its basic function and distribution, its ability to mediate gene expression, and the key working enzymes. Moreover, we introduced the role of DNA methylation in the development and differentiation of all types of T and B cells, discussed the controversial epigenomic differentiation models of T cells during memory development, and focused on discoveries of epigenetic control mechanisms in which the DNA methylation state is changed in both basic biological processes and the pathogenesis of a series of human autoimmune diseases. DNA methylation regulates the expression of genes that determine cell fates, predominantly via DNMTs. These genes encode key transcription factors, such as Ifng in Th1 and IL4 in Th2 cells and AICDA in GC B cells. By affecting related factor expression, dysregulated DNA methylation indirectly influences the regulatory networks in which these factors are involved, leading to amplification of effects and further deregulation of cell type-specific gene expression programmers. Although the important functions of DNA methylation in gene modification, cell differentiation and disease regulation have been confirmed, some questions still need to be clarified. For example, although it has been verified that DNA methylation plays an important role in globally controlling CD4^+^ memory differentiation, the function of individual DNMT or TET family members in directing or maintaining CD4^+^ T cell memory remains to be elucidated. In conclusion, DNA methylation is a promising field that links the roles of genetics, gene expression regulation, and environmental risk factors in autoimmune diseases. To beneficially give full play to the role of DNA methylation, comprehending the definite mechanisms and critical modifications of DNA methylation and discovering strategies to alter and achieve the desired magnitude and direction of immune responses, thereby providing a potential direction for better diagnosing, monitoring and treating the progression of diseases driven by epigenetics, is essential (Figure 1).

## Author Contributions

JL performed the literature search, wrote the first draft of the manuscript, and revised the manuscript. YW, GH, XL, and ZZ critically revised the manuscript and provided substantial scientific contribution. LL and ZX proposed the project and revised the manuscript. All authors approved the final version of the manuscript.

## Conflict of Interest

The authors declare that the research was conducted in the absence of any commercial or financial relationships that could be construed as a potential conflict of interest.

## Publisher’s Note

All claims expressed in this article are solely those of the authors and do not necessarily represent those of their affiliated organizations, or those of the publisher, the editors and the reviewers. Any product that may be evaluated in this article, or claim that may be made by its manufacturer, is not guaranteed or endorsed by the publisher.
